# In older adults undergoing surgical fixation or arthroplasty following upper limb fractures, does frailty predict post-operative complications and mortality? A systematic review and meta-analysis

**DOI:** 10.1016/j.xrrt.2026.100764

**Published:** 2026-04-30

**Authors:** Callum MacLeay, Jensen Murphy, Victoria Giglio, Aoife Leahy, Johan van der Stok, John Tristan Cassidy

**Affiliations:** a School of Medicine, University of Limerick, Limerick, Ireland; b University Hospital Limerick, HSE, Limerick, Ireland; c School of Allied Heath, Faculty of Education and Health Sciences, Ageing Research Centre, Health Research Institute University of Limerick, Limerick, Ireland; d Department of Ageing and Therapeutics, University Hospital Limerick, Limerick, Ireland; e Department of Trauma & Orthopaedic Surgery, University Hospital Limerick, Limerick, Ireland

**Keywords:** Frailty, Upper limb fracture, Modified frailty index, Orthopedic surgery, Post-operative complications, Treatment outcomes

## Abstract

**Background:**

There is growing recognition of the role of clinical frailty and its impact on health outcomes in aging cohorts. Frailty may be associated with mortality and complications following fracture; however, its impact on upper limb outcomes has yet to be quantified. This systematic review aims to evaluate the association between pre-operative frailty and post-operative mortality and complications in adults undergoing surgical treatment for upper limb fractures. Secondary outcomes included reoperation, adverse discharge destination and length of stay.

**Methods:**

MEDLINE, EMBASE, and Cochrane CENTRAL databases were searched on January 31, 2025, to identify studies that assessed pre-operative patient frailty and post-operative outcomes in adults following traumatic upper limb fractures. Data on 30-day mortality, post-operative complications, reoperation, hospital readmission, adverse discharge, Clavien-Dindo IV complications, and length of stay were extracted. Data were pooled using random-effects and fixed effects models for meta-analyses. Grading of Recommendations Assessment, Development and Evaluation assessment was used to assess the quality of available evidence.

**Results:**

Of the 4,013 records identified, 9 retrospective cohort studies were eligible for inclusion. Frailty was significantly associated with increased risk of 30-day mortality (odds ratio [OR] = 3.40, 95% confidence interval [CI] = 2.90-3.99, *P* < .001), post-operative complications (OR = 2.78, 95% CI = 1.99-3.88, *P* < .001), adverse discharge (OR = 2.96, 95% CI = 1.75-5.02, *P* < .001), and hospital readmission (OR = 3.73, 95% CI = 1.86-7.46, *P* < .001). No statistically significant association between frailty and reoperation was found (OR = 1.61, 95% CI = 1.36-1.91, *P* < .001). Grading of Recommendations Assessment, Development and Evaluation assessment found moderate certainty for post-operative complications and reoperation, and low to very low certainty for 30-day mortality, readmission, adverse discharge, Clavien-Dindo IV complications, and length of stay.

**Conclusion:**

Pre-operative frailty is associated with increased early mortality, post-operative complications, adverse discharge, and hospital readmission following surgical treatment for upper limb fractures in adults. Future prospective investigations are needed to standardize frailty screening and inform surgical decision-making in upper limb fractures.

Frailty is a clinical syndrome characterized by age-associated decline in multisystem physiological function.^19^ It significantly impairs immune response, metabolic regulation, and musculoskeletal strength, contributing to an increased risk of falls and poor recovery from physiological stressors.^4^^,^^21^ Approximately one-third of adults over age 65 experience a fall annually.^3^^,^^37^ Fractures, with upper limb fractures being among the most prevalent, occur in 10%-15% of these falls.^21^ Frailty is recognized as a key contributor to adverse surgical outcomes.^30^ It represents the body's inability to recover from surgical interventions and contributes to higher rates of mortality, post-operative complications, and prolonged hospital stays.^30^

Within orthopedic surgery, frailty has been associated with poor post-operative outcomes including malunion, displacement, and morbidity.^2^^,^^25^^,^^27^^,^^31^ Frailty can be assessed using various tools such as the 5-item modified frailty index (5-mFI), which assigns patients a frailty score based on common comorbidities,^10^ and the Clinical Frailty Scale (CFS), a pictorial scale from 1 to 9 based on a patient's functional status two weeks before assessment.^22^ Screening tools that assess frailty have been shown to predict worse outcomes in older adults.^22^ By 2050, adults over 65 are projected to make up 17% of the world's population, with frailty prevalence in this cohort reported as high as 43.7%.^11^^,^^39^ Given the rising incidence of upper limb fractures and the increasing demand for surgical interventions among older adults, understanding the relationship between frailty and post-operative outcomes is essential to guide clinical decision-making, optimize surgical outcomes, and preserve independence in this population.^37^^,^^39^

Orthopedic research on frailty has primarily focused on hip fractures and arthroscopy, and while frailty's role in lower limb fractures is well documented, its impact on upper limb fractures remains unknown.^17^ This systematic review aims to examine whether frailty is associated with adverse outcomes following surgical management of upper limb fractures. The primary aim of this study is to assess the association between frailty and post-operative mortality and complications. Secondary outcomes investigated are reoperation rates, readmission, adverse discharge, Clavien-Dindo grade IV complications, and extended length of stay (LOS).

## Materials and methods

### Literature search

This systematic review was conducted in accordance with the Preferred Reporting Items for Systematic Review and Meta-Analyses guidelines and was registered with PROSPERO (CRD420251035181). A search was performed on January 31, 2025, across MEDLINE, EMBASE, and Cochrane CENTRAL (1998-present). The search strategy utilized 60 terms under 5 medical indexing terms: ‘Frailty,’ ‘Fractures, Bone,’ ‘Orthopedic Procedures,’ ‘Treatment outcome,’ and ‘Adult’ (see Supplementary File S1 for full strategy). Following database import and deduplication, 2 reviewers independently screened titles, abstracts, and full texts using Rayyan's Systematic Review Software.^29^ Reference lists of included studies were screened for additional eligible studies as of April 2025.

### Eligibility criteria

Studies were eligible if they met the following criteria: (1) peer-reviewed, English-language articles; (2) participants aged ≥18 years with traumatic upper limb fractures involving the shoulder, elbow, and wrist joints (including fractures of the hand, radius, ulna, humerus, scapula, or clavicle) treated with surgical fixation or joint arthroplasty for an acute traumatic fracture as part of initial fracture management; (3) used a validated frailty assessment tool; and (4) reported outcomes concerning frailty, including post-operative complications, mortality, reoperation, readmission, adverse discharge, Clavien-Dindo IV complications, or LOS. The inclusion of all surgically treated upper limb fractures was intentional to reflect the full spectrum of upper limb trauma encountered in clinical practice. Exclusion criteria included: (1) nonprimary or non–peer-reviewed publications (eg, abstracts); (2) studies involving lower limb, spinal, or cranial fractures; (3) elective procedures unrelated to trauma; and (4) studies relying on unvalidated proxy frailty measures (eg, hypoalbuminemia, sarcopenia).

### Eligible frailty metrics

The 5-mFI, the 8-item modified frailty index (8-mFI), and the Hospital Frailty Risk Score (HFRS) were the only validated frailty measures included in this study. The 5-mFI is a common index in orthopedic surgery literature. It assesses 5 comorbidities: diabetes, congestive heart failure, hypertension requiring medication, chronic obstructive pulmonary disease or pneumonia, and nonindependence functional status.^10^ In accordance with previous validation, patients were considered frail if mFI ≥ 2.^15^^,^^36^ The 8-mFI uses the same criteria to assess frailty as the 5-mFI; however, it includes severe obesity (body mass index > 35), osteoporosis, and hypoalbuminemia (albumin <3.5 g/dL) as risk factors.^27^ Both the 5-mFI and 8-mFI are indices that quantify comorbidity burden and incorporate functional dependence to predict frailty and the risk of post-operative complications.^49^ This represents a notable distinction from traditional comorbidity indices, which primarily measure diagnosed disease burden without assessing functional status or diminished physiological reserve.^42^ In contrast, the HFRS captures frailty-related syndromes and health care utilization, providing a broader assessment of frailty beyond comorbidity alone.^13^ The HFRS is calculated as a weighted count of 109 frailty-related International Classification of Diseases, Tenth Revision diagnostic codes, where 0-5 points represent a low risk of frailty, 5-15 represent moderate, and >15 represent a severe risk (Table I).^40^^,^^43^ To ensure consistency across studies, mFI = 2 and the intermediate level (5-15 points) of the HFRS were used preferentially in data pooling as their definitions most aligned with mFI ≥2.^40^ Studies using continuous frailty scores were excluded from meta-analysis and synthesized narratively.

**Table I:** Five-mFI, 8-mFI, and HFRS frailty definitions.

Frailty measure	Scoring method	Frailty categories
Modified frailty index (5-mFI)	Count of 5 comorbidities (1 point each): History of diabetes mellitus History of congestive heart failure Hypertension requiring the use of medication History of chronic obstructive pulmonary disease Nonindependent functional status	0-1 = nonfrail **≥2 = frail**
Modified frailty index (8-mFI)	Count of 8 comorbidities (1 point each): History of diabetes mellitus History of congestive heart failure Hypertension requiring the use of medication History of chronic obstructive pulmonary disease Nonindependent functional status Severe obesity (BMI > 35) Diagnosis of osteoporosis Hypoalbuminemia (albumin < 3.5)	mFI 0 = no risk factors mFI 1 = 1-2 risk factors **mFI 2 = 3-4 risk factors** mFI 3 = 5+ risk factors
Hospital Frailty Risk Score (HFRS)	Weighted score based on 109 ICD-10 diagnostic codes (1 point each): Eg,: acute myocardial infarction, pleural effusion, dementia, heart failure, emphysema, angina, dizziness, senile cataracts, urinary incontinence, and unspecified fall	<5 = low risk **5-15 = intermediate risk** >15 = high risk

*HFRS*, Hospital Frailty Risk Score; *5-mFI*, 5-item modified frailty risk; *8-mFI*, 8-item modified frailty index; *BMI*, body mass index; *ICD-10*, International Classifications of Diseases, Tenth Revision.

Bolded categories indicate the frailty thresholds preferentially used for data pooling and cross-study comparison.

### Data extraction and quality assessment

Two reviewers independently extracted data using a standardized form designed for retrospective cohort studies. Extracted variables included study design, procedure type, demographics (sample size, age), frailty tool, outcomes, and reported effect sizes (odds ratios (ORs), confidence intervals (CIs), and *P* values). Primary outcomes, including post-operative complications (eg, wound, pulmonary, hematological, or renal events) and mortality (early post-operative death), were extracted. Secondary outcomes included reoperation rates, adverse discharge (defined as any discharge to a facility other than home, eg, rehabilitation center or skill nursing facility), readmission, LOS, and Clavien-Dindo grade IV complications. These are specifically life-threatening complications requiring intensive care unit management due to end organ dysfunction such as myocardial infarction, cardiac arrest, or septic shock.^10^^,^^33^^,^^51^ The STrengthening the Reporting of OBservational studies in Epidemiology checklist was utilized to assess study reporting quality and was analyzed descriptively to appraise the included studies.^9^ Methodological quality and risk of bias were assessed using the Newcastle-Ottawa Scale (NOS).^50^ The NOS evaluates 3 domains of cohort and case-control studies: selection, comparability, and outcome.^41^ Scores range from 0 to 9, with studies scoring ≥7 classified as high quality and low risk of bias.^47^ These results were used to assess the quality of the available evidence and the strength of resulting recommendations using the Grading of Recommendations Assessment, Development and Evaluation (GRADE) approach.^34^

### Data synthesis and analysis

Where possible, ORs and CIs were pooled for meta-analysis. To enable consistent comparison across studies, frailty variables were harmonized. Included studies reported frailty either as binary (frail vs. nonfrail) or as categorical levels (eg, low, intermediate, high). Short-term mortality was defined as post-operative death within 90 days. Meta-analyses were performed for outcomes reported by at least 3 studies using binary or categorical frailty classifications. Fixed-effects models were utilized when heterogeneity was low (I^2^ <50%) and random-effects models when heterogeneity was moderate-to-high (I^2^ ≥ 50%). Heterogeneity was assessed using I^2^, Cochran Q, and Tau^2^. Restricted maximum likelihood estimation was used to assess variance. Meta-regression analyses were conducted for outcomes with ≥5 studies, using the frailty tool, surgery type, geographic region, and publication year as moderators. Sensitivity analyses were performed using leave-one-out models. Publication bias was assessed using Egger regression test and a visual funnel plot inspection. All statistical analyses were performed using IBM SPSS Statistics, version 29.0 (IBM Corp., Armonk, NY).^20^

## Results

### Study screening

**Figure 1:**
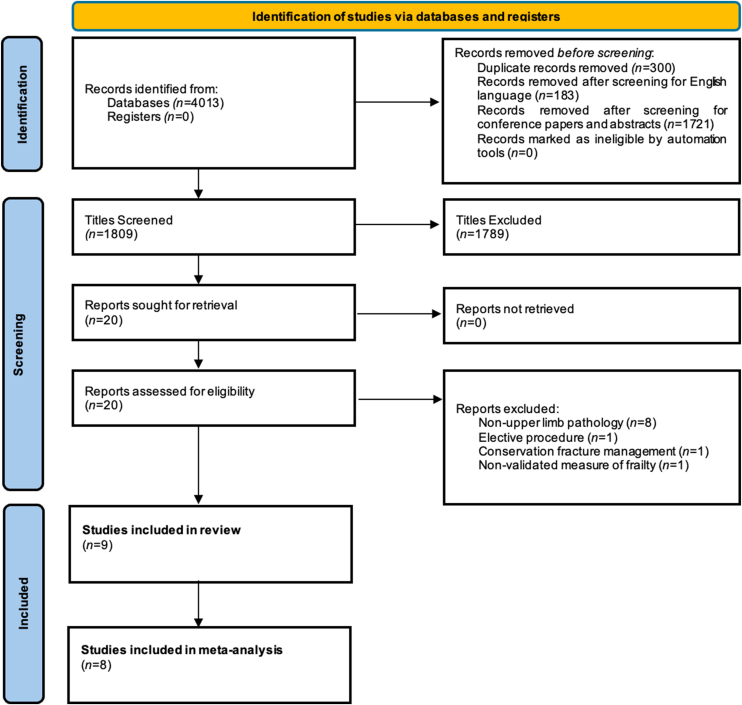
PRISMA flow diagram illustrating the study selection process. *PRISMA*, Preferred Reporting Items for Systematic Review and Meta-Analyses.

The database search identified 4,013 records. After removal of non-English studies, abstracts, and duplicates, 2,109 unique articles were screened by title and abstract. Twenty full texts were ultimately reviewed, and 9 studies met the inclusion criteria. The most common reason for full-text exclusion was surgical data related to nonupper limb pathologies (*n* = 8). Additional exclusions included unclear surgical indication (eg, elective total elbow arthroplasty), absence of operative management, and use of nonvalidated frailty measures (Fig. 1).

### Study characteristics

All 9 included studies were retrospective cohort studies published between 2018 and 2025. Eight studies were conducted in the United States of America and 7 used the American College of Surgeons National Surgical Quality Improvement Program database. Study cohorts ranged from 153 to 34,912 patients. Four studies focused on proximal humerus fractures, with varying interventions: open reduction internal fixation, arthroplasty, or both. Other fracture types included distal radius (*n* = 2), concurrent radius and ulna (*n* = 2), and distal humerus (*n* = 1). Frailty was most frequently assessed using the 5-mFI (*n* = 7). One study used the 8-mFI and 1 used the HFRS. Frailty was expressed categorically or as a binary variable, with mFI ≥2 indicating frailty. Two studies reported their frailty assessment categorically, while 6 reported their assessment as a binary variable. One study used the 5-mFI as a continuous frailty measure and was excluded from pooled analyses (Table II).

**Table II:** Characteristics of studies included in the meta-analysis.

Reference	Study design	Country	Frailty measure	Sample	Mean/median age (years)	Outcomes	Follow-up period
Congiusta et al^5^	Retrospective cohort	US	5-mFI	4,641	Mean: NR Range: 19-100 ≥65: 1,541 (33.2%)	Post-operative complications	30 d
						Rates of reoperation	
						Discharge outcomes	
Dave et al^7^	Retrospective cohort	US	5-mFI	5,654	Mean: 73.7	Post-operative complications	30 d
						Rates of reoperation	
Evans et al^10^	Retrospective cohort	US	5-mFI	2,004	Median: 66 (IQR: 59-74)	Post-operative complications	30 d
						Mortality	
						Length of stay	
						Rates of readmission	
						Rates of reoperation	
						Discharge outcomes	
						Clavien-Dindo IV complications	
Momtaz et al^27^	Retrospective cohort	US	8-mFI	22,313	Mean: 56 ± 16	Post-operative complications	30 d
						Length of stay	
						Rates of readmission	
						Rates of reoperation	
						Discharge outcomes	
						Clavien-Dindo IV complications	
Saltzman et al^33^	Retrospective cohort	US	5-mFI	846	Median: 66	Post-operative complications	30 d
						Mortality	
						Rates of reoperation	
						Rates of readmission	
						Discharge outcomes	
Spoden et al^40^	Retrospective cohort	Germany	HFRS	34,912	Mean: 75.0 ± 10.5	Post-operative complications	365 d
						Mortality	
						Rates of reoperation	
						Other complications (blood transfusion, trauma, intensive care)	
Wilson et al^51^	Retrospective cohort	US	5-mFI	6,423	Mean: 65.1 ± 9.6	Post-operative complications	30 d
						Length of stay	
						Rates of readmission	
						Rates of reoperation	
						Discharge outcomes	
						Clavien-Dindo IV complications	
Yi et al^53^	Retrospective cohort	US	5-mFI	3,893	Mean: age 68.0 ± 13.2	Post-operative complications	30 d
						Length of stay	
						Rates of reoperation	
						Mean operative time	
Zhang et al^54^	Retrospective cohort	US	5-mFI	153	Mean: 70	ASES	≥2 yr
						Rates of reoperation	
						Functional status	
						Pain scores	
						Range of motion	

*IQR*, interquartile range; *US*, United States of America; *mFI*, modified frailty index; *NR*, not reported; *ASES*, American Shoulder and Elbow Surgeons Shoulder.

### Outcomes and meta-analysis overview

Reported outcomes included reoperation (*n* = 9), post-operative complications (*n* = 7), discharge destination (*n* = 5), readmission (*n* = 4), mortality (*n* = 3), LOS (*n* = 4), and Clavien-Dindo IV complications (*n* = 3). For meta-analysis, we aligned categorical classifications with the binary threshold most frequently used across previous literature: mFI ≥ 2. Specifically, for Momtaz et al,^27^ we selected mFI = 2 vs. mFI = 0. Whereas for Spoden et al,^40^ we compared the intermediate-risk group (HFRS 5-15) to the low-risk group (HFRS < 5). This allowed us to pool comparable effect sizes across studies. One study, Yi et al,^53^ reported frailty as a continuous variable and was excluded from meta-analysis.

### Post-operative complications

**Figure 2:**
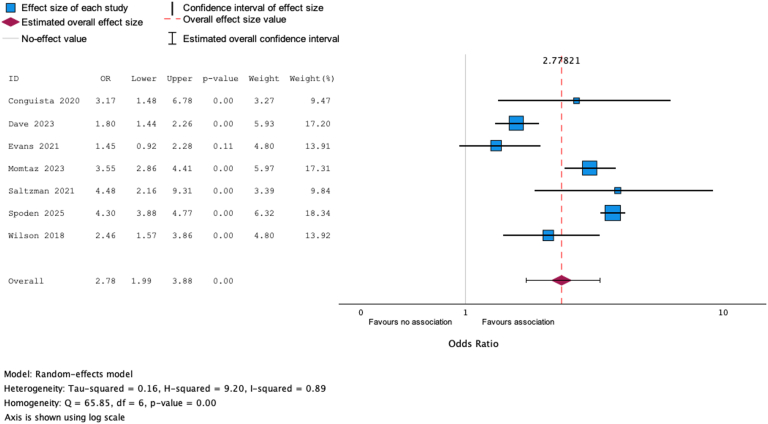
Association between pre-operative frailty and post-operative complications. Title: Frailty and post-operative complications forest plot. *OR*, odds ratio.

Seven studies examined post-operative complications using binary or categorical frailty classifications.^5^^,^^7^^,^^10^^,^^27^^,^^33^^,^^40^^,^^51^ Yi et al,^53^ which used continuous variables for frailty assessment, was excluded. Pooled analysis using a random-effects model demonstrated significantly increased odds of post-operative complications among frail patients (OR = 2.78, 95% CI = 1.99-3.88, *P* < .001) (Fig. 2). Egger test suggested potential publication bias (*P* = .032) and was supported by visual funnel plot asymmetry. Univariate meta-regression analyses were conducted to explore sources of heterogeneity (I^2^ = 89.1%). No significant moderation was observed for the frailty tool, surgery type, region, or publication year. Trends demonstrated increased odds of complications with the HFRS (OR = 1.92, 95% CI = 0.72-5.14) and 8-mFI (OR = 1.59, 95% CI = 0.57-4.40) compared to the 5-mFI; however, these differences were not statistically significant. A leave-one-out sensitivity analysis demonstrated that no individual study substantially altered the pooled effect estimate, and findings remained directionally consistent across all iterations.

### Mortality

**Figure 3:**
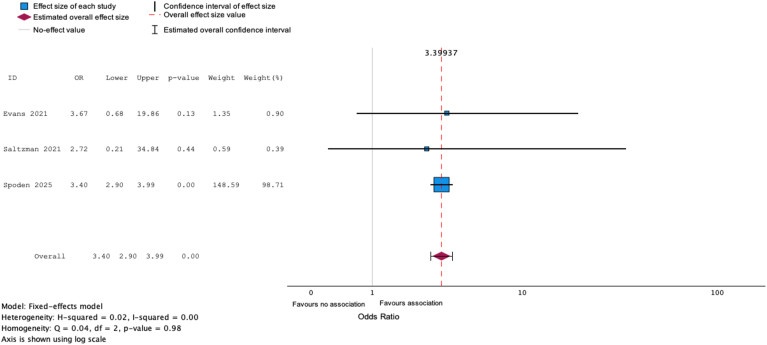
Association between pre-operative frailty and short-term mortality. Title: Frailty and short-term mortality forest plot. *OR*, odds ratio.

Three studies reported on short-term mortality. Two studies reported 30-day mortality,^10^^,^^33^ and 1 reported 90-day mortality.^40^ A fixed-effects model (I^2^ = 0%) demonstrated significantly increased odds of mortality in frail patients (OR = 3.40, 95% CI = 2.90-3.99, *P* < .001) (Fig. 3). Egger test indicated no evidence of publication bias (*P* = .056). A leave-one-out sensitivity analysis showed consistent findings across iterations (ORs: 3.35-3.40), with no evidence of heterogeneity. Meta-regression was not performed due to the limited study number and lack of between-study variance.

### Reoperation

Seven studies assessed reoperation risk. Six reported 30-day reoperation rates,^5^^,^^7^^,^^10^^,^^27^^,^^33^^,^^51^ and 1 study, Spoden et al,^40^ used a 1-year timeline. This study was retained due to conceptual alignment with the outcome, as reoperation reflects cumulative surgical failure, and frailty is a persistent risk factor. Zhang et al^54^ did not report reoperation rates as an OR, and Yi et al^53^ analyzed frailty data as a continuous variable. Therefore, both were excluded from the meta-analysis. Although 14% of patients underwent reoperation in this study, no significant association with mFI was reported (*P* = .80).^54^ Pooled analysis demonstrated a significant association between frailty and reoperation rates (OR = 1.61, 95% CI = 1.36-1.91, *P* < .001). Although statistical heterogeneity was moderate (I^2^ = 40.9%), a random-effects model was preferentially applied to account for methodological variation, including differences in follow-up duration and surgical approach. No publication bias was detected (Egger test *P* = .076). Frailty tool significantly moderated effect size (*P* = .034), explaining >99% of heterogeneity. A leave-one-out sensitivity analysis showed stability across all iterations with no single study disproportionately influencing the pooled effect estimate.

### Adverse discharge

**Figure 4:**
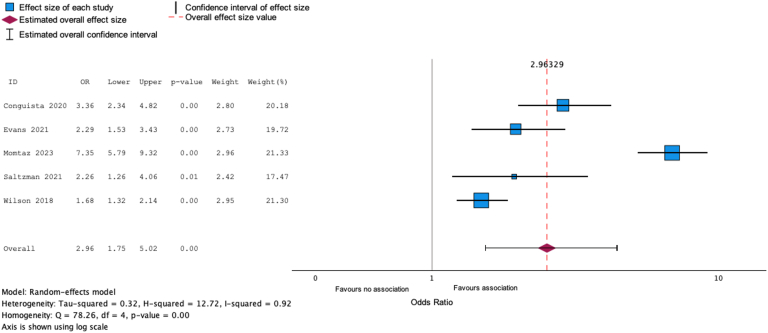
Association between pre-operative frailty and adverse discharge post-operatively. Title: Frailty and adverse discharge forest plot. *OR*, odds ratio.

Five studies examined this variable and showed considerable heterogeneity (I^2^ = 92%).^5^^,^^10^^,^^27^^,^^33^^,^^51^ A random-effects model found significantly higher odds of adverse discharge among frail patients (OR = 2.96, 95% CI = 1.75-5.02, *P* < .001) (Fig. 4). There was no significant evidence of publication bias (*P* = .166) according to Egger regression test. Univariate random-effects meta-regressions were conducted to explore moderators. The Frailty tool significantly influenced the effect size (*P* < .001), accounting for 78.5% of the heterogeneity between studies. Studies using the 5-mFI reported notably lower odds of adverse discharge compared to other tools (*P* = .040). Region and surgery type were not significant moderators. A leave-one-out sensitivity analysis showed consistent pooled ORs (2.29-3.14) across all iterations. The lowest effect size was observed when Momtaz et al^27^ was excluded, indicating that this study substantially contributed to the observed heterogeneity.

### Readmission

**Figure 5:**
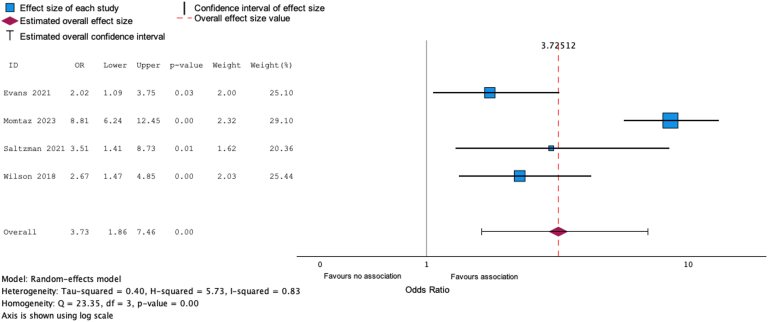
Association between pre-operative frailty and short-term readmission. Title: Frailty and readmission forest plot. *OR*, odds ratio.

Four studies reported on 30-day hospital readmission.^10^^,^^27^^,^^33^^,^^51^ Heterogeneity was substantial (I^2^ = 82.5%), and a random effects model was applied, showing a significant association between frailty and readmission rates (OR = 3.73, 95% CI = 1.86-7.46, *P* < .001) (Fig. 5). Egger test showed no significant evidence of publication bias (*P* = .140). Meta-regression was not conducted for the readmission outcome due to the small number of included studies. A leave-one-out sensitivity analysis showed consistently elevated pooled ORs (2.51-4.59). Heterogeneity was eliminated when Momtaz et al^27^ were excluded, suggesting a substantial contribution to overall heterogeneity.

### Clavien-Dindo IV complications

Three studies assessed Clavien-Dindo IV complications, showing significant between-study heterogeneity (I^2^ = 88.4%).^10^^,^^19^^,^^27^ A random-effects model demonstrated a nonsignificant association between Clavien-Dindo IV complications and frail patients (OR = 5.32, 95% CI = 0.32-88.32, *P* = .244). Egger test did not indicate significant evidence of publication bias (*P* = .857). A meta-regression could not be performed due to the small number of included studies. A leave-one-out sensitivity analysis revealed that the results were sensitive to individual studies. Exclusion of Evans et al^10^ significantly altered the effect estimate (OR = 29.76, 95% CI = 7.62-94.00) and eliminated heterogeneity.

### Length of stay

Four studies examined LOS following the surgical treatment of upper limb fractures.^10^^,^^27^^,^^51^^,^^53^ Only one, Momtaz et al^27^ reported LOS as a categorical variable with ORs. Patients with an mFI = 2 had significantly higher odds of experiencing prolonged hospitalizations (>3 days) compared to nonfrail patients (OR = 5.21, 95% CI = 4.29-6.33, *P* < .001). The remaining studies reported LOS as continuous or composite variables and could not be pooled. Evans et al^10^ and Wilson et al^51^ observed increasing LOS with frailty. A mFI ≥2 was associated with a median LOS of 2 days (interquartile range = 1-4) in Evans et al,^10^ and a mean LOS of 1.44 ± 12.9 days in Wilson et al^51^ In addition, Yi et al^53^ reported a mean LOS of 3.43 ± 7.29 days; however, LOS was not independently analyzed in their investigation. Due to heterogeneity in definitions, meta-analysis of LOS was not feasible.

### Quality of evidence and risk of bias

All studies provided adequate background information and rationale for their investigations and sufficiently described their participants. Most studies outlined their outcomes and provided a detailed description of the relevant variables in the utilized frailty assessments. Common pitfalls in reporting occurred in describing efforts to mitigate sources of bias and in describing the generalizability of results. Overall, most studies had a high quality of reporting and met the current standards of reporting of observational studies. Quality of reporting for included studies is presented in Supplementary Table S1.

Methodological quality and risk of bias were assessed using the NOS. Results are reported in Supplementary Table S2. Eight of 9 studies scored ≥ 8, indicating high quality of evidence of the included studies. One study, Zhang et al,^54^ scored 5, failing to adjust for confounders or make complete statistical adjustments. Most commonly, included studies lost one point for attrition bias.

As all included studies were of retrospective cohort design, they inherently provided a low certainty of evidence. Based on the GRADE criteria, the certainty of evidence was moderate for post-operative complications and reoperation, but low to very low for mortality, hospital readmission, Clavien-Dindo IV complications, and LOS. The certainty of evidence for these aforementioned outcomes was downgraded due to inconsistency and imprecision.

## Discussion

The most important finding of this review was that pre-operative frailty was associated with increased risk of 30-day mortality and early post-operative complications among adults undergoing surgical management for upper limb fractures. Frail patients were over 3 times as likely to experience short-term mortality and nearly 3 times as likely to develop complications compared to their nonfrail counterparts. Frailty was also associated with increased odds of adverse discharge and unplanned hospital readmission. Unlike traditional risk factors such as age or isolated comorbidities, frailty reflects diminished physiological reserve and vulnerability to surgical intervention.^4^ By integrating multisystem decline and functional impairment, frailty may capture operative risk not fully reflected by conventional comorbidity measures. While prior meta-analyses have explored the surgical outcomes of frail patients within general orthopedics and hip fracture repair, this is the first review, to our knowledge, to specifically synthesize data on upper limb fractures. These findings underscore the importance of incorporating frailty assessment into pre-operative planning and discharge decision-making for patients undergoing surgical management of upper limb fractures.

The association between frailty and 30-day mortality and post-operative complications observed in this study aligns with existing evidence across various orthopedic populations. A recent meta-analysis across all orthopedic subspecialities identified frailty as an independent predictor of 30-day mortality (OR = 2.89, 95% CI = 2.00-4.18).^17^ Within hip fracture cohorts, prior meta-analyses reported a two- to four-fold increased risk of 30-day mortality among frail adults undergoing surgical treatment of hip fractures.^12^^,^^16^ In terms of absolute mortality, 1-year mortality is commonly reported between 20% and 30%, increasing to almost 50% by 3 years in older adults.^8^^,^^46^ Although direct comparisons between all upper limb fracture types are limited,^38^ proximal humerus fracture population studies report clinically relevant mortality rates, with 1-year mortality of 10%-12% in patients aged ≥65 years and approximately 33% at 4 years.^6^^,^^46^ Mortality also varies by treatment modality, with nonoperative treatment having the highest 1 year mortality rate at 16.4%, which may reflect selection bias whereby frailer individuals are preferentially managed with nonoperative care.^48^ Although absolute mortality following proximal humerus fractures remains comparatively lower than in hip fracture cohorts, rates are clinically significant, indicating that frailty confers meaningful perioperative risk in fracture populations traditionally perceived as lower risk.

With respect to post-operative complications, a systematic review of frail patients with hip fractures reported lower odds of post-operative complications than observed in our analysis (OR = 1.46, 95% CI = 1.13-1.90).^39^ This difference may reflect variation in fracture patterns, surgical complexity, or perioperative care pathways between upper limb and hip fracture populations. Our review intentionally included fractures of the wrist, elbow, and shoulder to capture the full spectrum of surgically treated upper limb trauma encountered in clinical practice. This inclusive approach enhances external validity and reduces selection bias based on perceived fracture severity, but may also introduce greater heterogeneity in operative techniques and complications. Collectively, these findings reinforce that frailty is a consistent predictor of post-operative complications across orthopedic trauma populations, irrespective of fracture location.

Beyond immediate surgical risks, frailty was also associated with adverse discharge and unplanned readmissions. Our analysis revealed a nearly threefold increased odds of adverse discharge for frail patients, surpassing results from prior studies in lower limb fractures. A large study utilizing the 5-mFI in a cohort of hip fracture patients reported a modest increase in odds of adverse discharge (OR = 1.23, 95% CI = 1.18-1.28),^23^ while another study employing a 6-item mFI found that frail patients from a lower extremity fracture cohort were more than twice as likely to experience adverse discharge (OR = 2.09, 95% CI = 1.84-2.37).^32^ In a study of general orthopedic fracture patients, 94.4% of frail individuals (*n* = 73) were discharged to rehabilitation hospitals or skilled nursing facilities.^14^ In contrast, the studies in our review provided limited information regarding the discharge destinations of their frail patients following upper limb fracture surgery. Future research should aim to characterize the discharge disposition of upper limb fracture patients to better recognize the impact of frailty on post-operative outcomes.

Regarding readmission, our review found almost a 4 times increased risk following the surgical treatment of upper limb fractures. Interestingly, in our cohort of upper limb fracture patients, readmission rates were over two-fold higher compared to hip fracture patients from a systematic review by Ma et al^24^ (RR = 1.63, 95% CI = 1.29-2.06).^24^ In addition, Wong et al^52^ reported no significant association between frail hip fracture patients and 30-day readmission.^52^ This nonsignificant difference was attributed to improved inpatient and transition care to rehabilitation centers for hip fracture patients.^52^ The discrepancy between our results and previous reports in the hip fracture population may reflect less comprehensive inpatient and community step-down care for upper limb fracture patients compared to lower limb fracture patients. This underscores that upper limb fractures in frail patients carry significant perioperative risk and supports the need to evaluate and optimize post-operative management in this population.

This review incorporated studies using several different frailty tools, reflecting a lack of standardization in frailty assessment within orthopedics.^35^ The 5-mFI was most frequently utilized and is favored in orthopedics for various reasons. It is the fastest to administer, the easiest to commit to memory, and therefore more likely to be applied by orthopedic surgeons in busy clinical settings.^1^^,^^33^ Compared to the broader 11-mFI, the 5-mFI offers similar predictive validity for surgical outcomes and requires fewer data points, thus reducing missing data and improving feasibility in retrospective analyses.^44^^,^^51^ However, the question remains whether abbreviated tools like the 5-mFI truly reflect clinical frailty rather than comorbidity. Although frailty indices such as the mFI incorporate comorbid conditions, frailty and comorbidity are conceptually distinct. Comorbidity measures are commonly used to quantify the burden of disease by counting chronic conditions,^28^ whereas frailty scoring systems assess comorbidities alongside functional dependence to approximate frailty and reflect a patient's vulnerability to adverse outcomes.^4^ Importantly, counting diseases alone does not account for disease severity, functional impairment, or physiological vulnerability that may exist independent of disease count.^42^ To address the limitations of disease count–based comorbidity indices, other authors advocate for the inclusion of tools with a minimum of 30 multidomain variables to provide a more robust and comprehensive assessment of a patient frailty, such as the CFS.^18^^,^^26^^,^^35^ Subramaniam et al^45^ found poor agreement between the CFS and a 10-item mFI, where the CFS was an independent predictor of long-term survival in intensive care unit patients and the 10-mFI better correlated with comorbidities.^45^ To date, no studies have directly compared the abbreviated 5-mFI to the CFS in orthopedic cohorts. Despite these limitations, the 5-mFI is widely used in orthopedics clinics, polytrauma situations, and resource-limited environments due to its feasibility and simplicity.^1^^,^^17^^,^^33^ Further prospective research is needed to validate its performance across diverse orthopedic populations and against comprehensive frailty assessment tools that are more sensitive to frailty rather than morbidity. The possible role of functionally based frailty scores, such as the CFS, in pre-operative planning for upper limb trauma and orthopedic surgery patients is unknown and has yet to be explored.

This systematic review has numerous strengths, including a comprehensive search strategy, rigorous methodological approach, and a transparent assessment of study quality. However, this study also has notable limitations. First, all included studies were retrospective cohorts, which are inherently limited by potential selection bias and misclassification. Second, most studies relied on administrative databases. These can minimize clinical details and contextual factors that affect patient frailty status and post-operative outcomes. Third, substantial heterogeneity was observed in several outcomes, including post-operative complications (I^2^ = 89.1%), adverse discharge (I^2^ = 92%), and hospital readmission (I^2^ = 82.5%). The heterogeneity may reflect differences in the application of frailty tools, fracture patterns, and surgical techniques. Reliable fracture-specific subgroup meta-analysis was not feasible due a limited number of studies within fracture subtypes. In addition, the timing of surgical intervention was not uniformly reported across included studies and may have varied according to institutional policy, outpatient triaging, operating room availability, or medical optimization. Delays in operative management could influence post-operative outcomes, particularly in frail patients, and represent a potential source of heterogeneity. Therefore, the effect estimates should be interpreted with caution, as demonstrated by our GRADE assessment. Finally, despite the use of validated frailty measures in every study, uncertainty remains as to whether these tools accurately reflect clinical frailty rather than comorbidity. Many database-derived frailty indices rely on diagnostic codes that overlap with traditional comorbidity measures and may under-represent functional and cognitive vulnerability. Thus, some observed associations may represent multimorbidity rather than physiological frailty. Further research should prioritize prospective study designs and the use of standardized, clinically assessed frailty measures to better distinguish frailty from comorbidity and explore the integration of frailty screening tools into routine pre-operative assessment.

## Conclusion

Pre-operative frailty is associated with increased early mortality, post-operative complications, adverse discharge, and hospital readmission following the surgical treatment of upper limb fractures in the adult population. These findings show frailty to be an important pre-operative risk factor that should be considered for patients undergoing surgery for upper limb fractures. Given the growing burden of frailty in aging populations, future studies are needed to establish standardized frailty screening tools that can be used to assess operative risk and inform surgical decision-making when treating upper limb fractures.

## Disclaimers:

Funding: No funding was disclosed by the authors.

Conflicts of interest: The authors, their immediate families, and any research foundations with which they are affiliated have not received any financial payments or other benefits from any commercial entity related to the subject of this article.
