# Supplementary material for: In older adults undergoing surgical fixation or arthroplasty following upper limb fractures, does frailty predict post-operative complications and mortality? A systematic review and meta-analysis
Source: JSES Rev Rep Tech. 2026 Apr 30;6(3):100764. doi: 10.1016/j.xrrt.2026.100764 (PMC13265995; doi:10.1016/j.xrrt.2026.100764)
Supplement: Supplementary File S1 [file mmc1.docx]

**Research Q:** In older adults undergoing surgical fixation or arthroplasty following upper limb fractures, does frailty predict post-operative complications and mortality?

**Search Strategies:**

### Appendix I.I OVID MEDLINE Search Strategy

| [# ▲](http://ovidsp.tx.ovid.com/sp-3.27.0b/ovidweb.cgi?&S=FBCBFPJHNEDDABABNCGKOGOBBFLJAA00&Sort+Sets=descending) | **Searches** |
| --- | --- |
| 1 | exp **Frailty/** |
| 2 | Frail*.mp |
| 3 | Frailty index.mp. |
| 4 | (Frailty Phenotype or Fried Criteria).mp. |
| 5 | Frailty Indicator.mp. |
| 6 | Frail* Scale.mp. |
| 7 | Short Physical Performance Battery.mp. |
| 8 | Physical frailty.mp |
| 9 | (Pre-operative or Perioperative frail*).mp |
| 10 | exp Geriatric Assessment/ |
| 11 | 1 or 2 or 3 or 4 or 5 or 6 or 7 or 8 or 9 or 10 |
| 12 | **Exp Fractures, Bone/** |
| 13 | Exp Upper Extremity/ |
| 14 | Upper Limb.mp |
| 15 | Exp Arm Injuries/ |
| 16 | Arm fracture.mp |
| 17 | Forearm fracture.mp. |
| 18 | Fracture.mp |
| 19 | 12 or 13 or 14 or 15 or 16 or 17 or 18 |
| 20 | **Exp Orthopedic Procedures/** |
| 21 | **Orthop*dic Procedures.mp.** |
| 22 | Exp Orthopedics/ |
| 23 | Orthop*edics.mp. |
| 24 | Orthop*edic Surg* |
| 25 | Exp Surgical Procedures, Operative/ |
| 26 | Exp Fracture Fixation, Internal / |
| 27 | External Fixation.mp. |
| 28 | Surg* |
| 29 | 20 or 21 or 22 or 23 or 24 or 25 or 26 or 27 or 28 or 29 |
| 30 | **Exp Treatment outcome/** |
| 31 | Patient Outcome*.mp. |
| 32 | Functional outcome*.mp. |
| 33 | Clinical outcome*.mp. |
| 34 | Exp Patient-reported outcome measures/ |
| 35 | PROM*.mp |
| 36 | Exp Mortality/ |
| 37 | Exp “Recovery of function”/ |
| 38 | Exp “Quality of Life”/ |
| 39 | Exp “Length of Stay”/ |
| 40 | Exp Hospitalization/ |
| 41 | Exp Continuity of Patient Care/ |
| 42 | Exp “Activities of daily living”/ |
| 43 | Exp Motion therapy, Continuous Passive/ |
| 44 | Exp Pain management/ |
| 45 | Exp Postoperative care/ |
| 46 | Exp Post-operative complications/ |
| 47 | Adverse outcome*.mp. |
| 48 | Exp Surgical Wound Infection/ |
| 49 | Exp Fractures, Ununited/ |
| 50 | Delayed healing.mp. |
| 51 | Malunited fractures.mp. |
| 52 | Hardware failure.mp. |
| 53 | Exp Reoperation/ |
| 54 | Adverse event*.mp. |
| 55 | Exp Fracture healing/ |
| 56 | 30 or 31 or 32 or 33 or 34 or 35 or 36 or 37 or 38 or 39 or 40 or 41 or 42 or 43 or 44 or 45 or 46 or 47 or 48 or 49 or 50 or 51 or 52 or 53 or 54 or 55 |
| 57 | **Exp Adult/** |
| 58 | Exp Geriatrics/ |
| 59 | Exp aging/ |
| 60 | Elderly.mp |
| 61 | 57 or 58 or 59 or 60 |
| 62 | 11 and 25 and 33 and 46 and 55 and 60 |
| 63 | limit 61 to (English language) |
| 64 | Limit 62 to full text |

Green = Frailty terms

Orange = Upper Limb Fracture Terms

Yellow = Surgical terms

Purple = clinical outcome terms

Light Blue = Adult population terms

Dark Blue = limits

### Appendix I.II EMBASE Search Strategy

| [# ▲](http://ovidsp.tx.ovid.com/sp-3.27.0b/ovidweb.cgi?&S=FBCBFPJHNEDDABABNCGKOGOBBFLJAA00&Sort+Sets=descending) | **Searches** |
| --- | --- |
| 1 | **‘frailty’/exp Or ‘frailty’** |
| 2 | ‘frail*’ |
| 3 | ‘frailty index’ |
| 4 | ‘frailty phenotype’ OR ‘fried criteria’ |
| 5 | ‘frailty indicator’ |
| 6 | ‘frail* scale’ |
| 7 | ‘short physical performance battery’ |
| 8 | ‘physical frailty’ |
| 9 | ‘pre-operative OR perioperative frail*’ |
| 10 | ‘geriatric assessment’/exp |
| 11 | 1 or 2 or 3 or 4 or 5 or 6 or 7 or 8 or 9 or 10 |
| 12 | **‘bone fractures’/exp** |
| 13 | ‘upper extremity’/exp |
| 14 | ‘Upper Limb’ |
| 15 | ‘Arm injuries’/exp |
| 16 | ‘Arm fracture’ |
| 17 | ‘Forearm fracture’ |
| 18 | ‘fracture’ |
| 19 | 12 or 13 or 14 or 15 or 16 or 17 or 18 |
| 20 | **‘orthopedic procedures’/exp** |
| 21 | ‘Orhop*dic procedures’ |
| 22 | ‘orthopedics’/exp |
| 23 | ‘Orthop*dics’ |
| 24 | ‘orthop*edic surg*’ |
| 25 | ‘surgical procedures, operative’/exp |
| 26 | ‘fracture fixation, internal’/exp |
| 27 | ‘external fixation’ |
| 28 | ‘surg*’ |
| 29 | 20 or 21 or 22 or 23 or 24 or 25 or 26 or 27 or 28 |
| 30 | **‘treatment outcome’/exp** |
| 31 | ‘patient outcome*’ |
| 32 | ‘functional outcome*’ |
| 33 | ‘clinical outcome*’ |
| 34 | ‘patient-reported outcome measures’/exp |
| 35 | ‘prom*’ |
| 36 | ‘mortality’/exp |
| 37 | ‘recovery of function’/exp |
| 38 | ‘quality of life’/exp |
| 39 | ‘length of stay’/exp |
| 40 | ‘hospitalization’/exp |
| 41 | ‘continuity of patient care’/exp |
| 42 | ‘activities of daily living”/exp |
| 43 | ‘motion therapy, continuous passive’/exp |
| 44 | ‘pain management’/exp |
| 45 | ‘postoperative care’/exp |
| 46 | ‘post-operative complications’/exp |
| 47 | ‘adverse outcome*’ |
| 48 | ‘surgical wound infection’/exp |
| 49 | ‘fractures, ununited’/exp |
| 50 | ‘delayed healing’ |
| 51 | ‘fractures, malunited’ |
| 52 | ‘hardware failure’ |
| 53 | ‘reoperation’/exp |
| 54 | ‘adverse event’ |
| 55 | ‘fracture healing’/exp |
| 56 | 30 or 31 or 32 or 33 or 34 or 35 or 36 or 37 or 38 or 39 or 40 or 41 or 42 or 43 or 44 or 45 or 46 or 47 or 48 or 49 or 50 or 51 or 52 or 53 or 54 or 55 |
| 57 | **‘adult’/exp** |
| 58 | ‘geriatrics’/exp |
| 59 | ‘aging’/exp |
| 60 | ‘elderly’ |
| 61 | 57 or 58 or 59 or 60 |
| 62 | 11 and 19 and 29 and 56 and 61 |
| 63 | #62 AND [english]/lim |
| 64 | #62 AND [english]/lim AND ([embase]/lim OR [preprint]/lim OR [pubmed-not-medline]/lim) |
| 65 | #62 AND [english]/lim AND ([embase]/lim OR [preprint]/lim OR [pubmed-not-medline]/lim) AND ([article]/lim OR [article in press]/lim OR [data papers]/lim OR [preprint]/lim) |

Green = Frailty terms

Orange = Upper Limb Fracture Terms

Yellow = Surgical terms

Purple = clinical outcome terms

Light Blue = Adult population terms

Dark Blue = limits

### Appendix I.III Cochrane CENTRAL and CDSR Search Strategy

| [# ▲](http://ovidsp.tx.ovid.com/sp-3.27.0b/ovidweb.cgi?&S=FBCBFPJHNEDDABABNCGKOGOBBFLJAA00&Sort+Sets=descending) | **Searches** |
| --- | --- |
| 1 | **MeSH descriptor: [Frailty] explode all trees** |
| 2 | (Frail*) (Word variations have been searched) |
| 3 | (Frailty index) (Word variations have been searched) |
| 4 | (Frailty Phenotype or Fried Criteria) (Word variations have been searched) |
| 5 | (Frailty Indicator) (Word variations have been searched) |
| 6 | (Frail* Scale) (Word variations have been searched) |
| 7 | (Short Physical Performance Battery) (Word variations have been searched) |
| 8 | (Physical frailty) (Word variations have been searched) |
| 9 | (Pre-operative or Perioperative frail*) (Word variations have been searched) |
| 10 | MeSH descriptor: [Geriatric Assessment] explode all trees |
| 11 | #1 OR #2 OR #3 OR #4 OR #5 OR #6 OR #7 OR #8 OR #9 OR #10 |
| 12 | **MeSH descriptor: [Fractures, Bone] explode all trees** |
| 13 | MeSH descriptor: [Upper Extremity] explode all trees |
| 14 | (Upper Limb) (Word variations have been searched) |
| 15 | MeSH descriptor: [Arm Injuries] explode all trees |
| 16 | (Arm fracture) (Word variations have been searched) |
| 17 | (Forearm fracture) (Word variations have been searched) |
| 18 | (Fracture) (Word variations have been searched) |
| 19 | #12 OR #13 OR #14 OR #15 OR #16 OR #17 OR #18 OR #19 |
| 20 | **MeSH descriptor: [Orthopedic Procedures] explode all trees** |
| 21 | (Orthop*dic Procedures) (Word variations have been searched) |
| 22 | MeSH descriptor: [Orthopedics] explode all trees |
| 23 | (Orthop*dics) (Word variations have been searched) |
| 24 | (Orthop*edic Surg*) (Word variations have been searched) |
| 25 | MeSH descriptor: [Surgical Procedures, Operative] explode all trees |
| 26 | MeSH descriptor: [Fracture Fixation, Internal] explode all trees |
| 27 | (External Fixation) (Word variations have been searched) |
| 28 | (Surg*) (Word variations have been searched) |
| 29 | #20 OR #21 OR #22 OR #23 OR #24 OR #25 OR #26 OR #27 OR #28 |
| 30 | **MeSH descriptor: [Treatment outcome] explode all trees** |
| 31 | (Patient outcome*) (Word variations have been searched) |
| 32 | (Functional outcome) (Word variations have been searched) |
| 33 | (Clinical outcome) (Word variations have been searched) |
| 34 | MeSH descriptor: [Patient-reported outcome measures] explode all trees |
| 35 | (PROM*) (Word variations have been searched) |
| 36 | MeSH descriptor: [Mortality] explode all trees |
| 37 | MeSH descriptor: [Recovery of Function] explode all trees |
| 38 | MeSH descriptor: [Quality of Life] explode all trees |
| 39 | MeSH descriptor: [Length of Stay] explode all trees |
| 40 | MeSH descriptor: [Hospitalization] explode all trees |
| 41 | MeSH descriptor: [Continuity of Patient Care] explode all trees |
| 42 | MeSH descriptor: [Activities of Daily Living] explode all trees |
| 43 | MeSH descriptor: [Motion therapy, Continuous Passive] explode all trees |
| 44 | MeSH descriptor: [Pain Management] explode all trees |
| 45 | MeSH descriptor: [Postoperative care] explode all trees |
| 46 | **MeSH descriptor: [Postoperative Complications] explode all trees** |
| 47 | (Adverse Outcome*) (Word variations have been searched) |
| 48 | MeSH descriptor: [Surgical Wound Infection] explode all trees |
| 49 | MeSH descriptor: [Fractures, Ununited] explode all trees |
| 50 | (Delayed healing) (Word variations have been searched) |
| 51 | (Fractures, Malunited) (Word variations have been searched) |
| 52 | (Hardware failure) (Word variations have been searched) |
| 53 | MeSH descriptor: [Reoperation] explode all trees |
| 54 | (Adverse event*) (Word variations have been searched) |
| 55 | MeSH descriptor: [Fracture healing] explode all trees |
| 56 | #30 OR #31 OR #32 OR #33 OR #34 OR #35 OR #36 OR #37 OR #38 OR #39 OR #40 OR #41 OR #42 OR #43 OR #44 OR #45 OR #46 OR #47 OR #48 OR #49 OR #50 OR #51 OR #52 OR #53 OR #54 OR #55 |
| 57 | **MeSH descriptor: [Adult] in all MeSH products** |
| 58 | MeSH descriptor: [Geriatrics] explode all trees |
| 59 | MeSH descriptor: [Aging] explode all trees |
| 60 | (Elderly) (Word variations have been searched) |
| 61 | #57 OR #58 OR #59 OR #60 |
| 62 | #11 AND #19 AND #29 AND #56 AND #61 |
| 63 | #62 AND English:la |
| 64 | Limit 62 to full text |

Green = Frailty terms

Orange = Upper Limb Fracture Terms

Yellow = Surgical terms

Purple = clinical outcome terms

Light Blue = Adult population terms

Dark Blue = limits
