# Supplementary material for: In older adults undergoing surgical fixation or arthroplasty following upper limb fractures, does frailty predict post-operative complications and mortality? A systematic review and meta-analysis
Source: JSES Rev Rep Tech. 2026 Apr 30;6(3):100764. doi: 10.1016/j.xrrt.2026.100764 (PMC13265995; doi:10.1016/j.xrrt.2026.100764)
Supplement: Supplementary Table S1 [file mmc2.docx]

**Table I.** 5-mFI, 8-mFI and HFRS Frailty Definitions

| Frailty Measure | Scoring Method | Frailty Categories |
| --- | --- | --- |
| Modified Frailty Index (5-mFI) | Count of 5 comorbidities (1 point each):  History of diabetes mellitus  History of congestive heart failure  Hypertension requiring the use of medication  History of chronic obstructive pulmonary disease  Non-independent functional status | 0–1 = Non-frail **≥2 = Frail** |
| Modified Frailty Index (8-mFI) | Count of 8 comorbidities (1 point each):  History of diabetes mellitus  History of congestive heart failure  Hypertension requiring the use of medication  History of chronic obstructive pulmonary disease  Non-independent functional status  Severe obesity (BMI>35)  Diagnosis of osteoporosis  Hypoalbuminemia (albumin<3.5) | mFI 0 = No risk factors mFI 1 = 1-2 risk factors  **mFI 2 = 3-4 risk factors**  mFI 3 = 5+ risk factors |
| Hospital Frailty Risk Score (HFRS) | Weighted score based on 109 ICD-10 diagnostic codes (1 point each):  E.g.: Acute myocardial infarction, pleural effusion, dementia, heart failure, emphysema, angina, dizziness, senile cataracts, urinary incontinence, unspecified fall | <5 = Low risk **5–15 = Intermediate risk** >15 = High risk |

BMI=Body Mass Index, ICD-10 =International Classitfications of Diseases 10^th^ revision
