# Supplementary material for: In older adults undergoing surgical fixation or arthroplasty following upper limb fractures, does frailty predict post-operative complications and mortality? A systematic review and meta-analysis
Source: JSES Rev Rep Tech. 2026 Apr 30;6(3):100764. doi: 10.1016/j.xrrt.2026.100764 (PMC13265995; doi:10.1016/j.xrrt.2026.100764)
Supplement: Supplementary Table S2 [file mmc3.docx]

**Table II.** Characteristics of studies included in the meta-analysis

| Reference | Study design | Country | Frailty Measure | Sample | Mean/Median age (years) | Outcomes | Follow-up period |
| --- | --- | --- | --- | --- | --- | --- | --- |
| Congiusta et al.^5^ | Retrospective cohort | US | 5-mFI | 4641 | Mean: NR Range: 19-100  ≥ 65: 1541 (33.2%) | Post-op complications | 30 days |
|  |  |  |  |  |  | Rates of reoperation |  |
|  |  |  |  |  |  | Discharge outcomes |  |
| Dave et al.^7^ | Retrospective cohort | US | 5-mFI | 5654 | Mean: 73.7 | Post-op complications | 30 days |
|  |  |  |  |  |  | Rates of reoperation |  |
| Evans et al.^10^ | Retrospective cohort | US | 5-mFI | 2004 | Median: 66 (IQR: 59-74) | Post-op complications | 30 days |
|  |  |  |  |  |  | Mortality |  |
|  |  |  |  |  |  | Length of Stay |  |
|  |  |  |  |  |  | Rates of readmission |  |
|  |  |  |  |  |  | Rates of reoperation |  |
|  |  |  |  |  |  | Discharge outcomes |  |
|  |  |  |  |  |  | Clavien-Dindo IV complications |  |
| Momtaz et al.^27^ | Retrospective cohort | US | 8-mFI | 22313 | Mean: 56 ± 16 | Post-op complications | 30 days |
|  |  |  |  |  |  | Length of Stay |  |
|  |  |  |  |  |  | Rates of readmission |  |
|  |  |  |  |  |  | Rates of reoperation |  |
|  |  |  |  |  |  | Discharge outcomes |  |
|  |  |  |  |  |  | Clavien-Dindo IV complications |  |
| Saltzman et al.^33^ | Retrospective cohort | US | 5-mFI | 846 | Median: 66 | Post-op complications | 30 days |
|  |  |  |  |  |  | Mortality |  |
|  |  |  |  |  |  | Rates of reoperation |  |
|  |  |  |  |  |  | Rates of readmission |  |
|  |  |  |  |  |  | Discharge outcomes |  |
| Spoden et al.^40^ | Retrospective cohort | Germany | HFRS | 34912 | Mean: 75.0 ± 10.5 | Post-op complications | 365 days |
|  |  |  |  |  |  | Mortality |  |
|  |  |  |  |  |  | Rates of reoperation |  |
|  |  |  |  |  |  | Other complications (blood transfusion, trauma, intensive care) |  |
| Wilson et al.^50^ | Retrospective cohort | US | 5-mFI | 6423 | Mean: 65.1 ± 9.6 | Post-op complications | 30 days |
|  |  |  |  |  |  | Length of Stay |  |
|  |  |  |  |  |  | Rates of readmission |  |
|  |  |  |  |  |  | Rates of reoperation |  |
|  |  |  |  |  |  | Discharge outcomes |  |
|  |  |  |  |  |  | Clavien-Dindo IV complications |  |
| Yi et al.^52^ | Retrospective cohort | US | 5-mFI | 3893 | Mean: age 68.0 ± 13.2 | Post-op complications | 30 days |
|  |  |  |  |  |  | Length of Stay |  |
|  |  |  |  |  |  | Rates of reoperation |  |
|  |  |  |  |  |  | Mean operative time |  |
| Zhang  et al.^53^ | Retrospective cohort | US | 5-mFI | 153 | Mean: 70 | ASES | ≥ 2 years |
|  |  |  |  |  |  | Rates of reoperation |  |
|  |  |  |  |  |  | Functional Status |  |
|  |  |  |  |  |  | Pain scores |  |
|  |  |  |  |  |  | Range of Motion |  |

US = United States of America, mFI = Modified Frailty Index, NR = Not reported, Post-op = Post-operative, ASES = American Shoulder and Elbow Surgeons Shoulder Score
